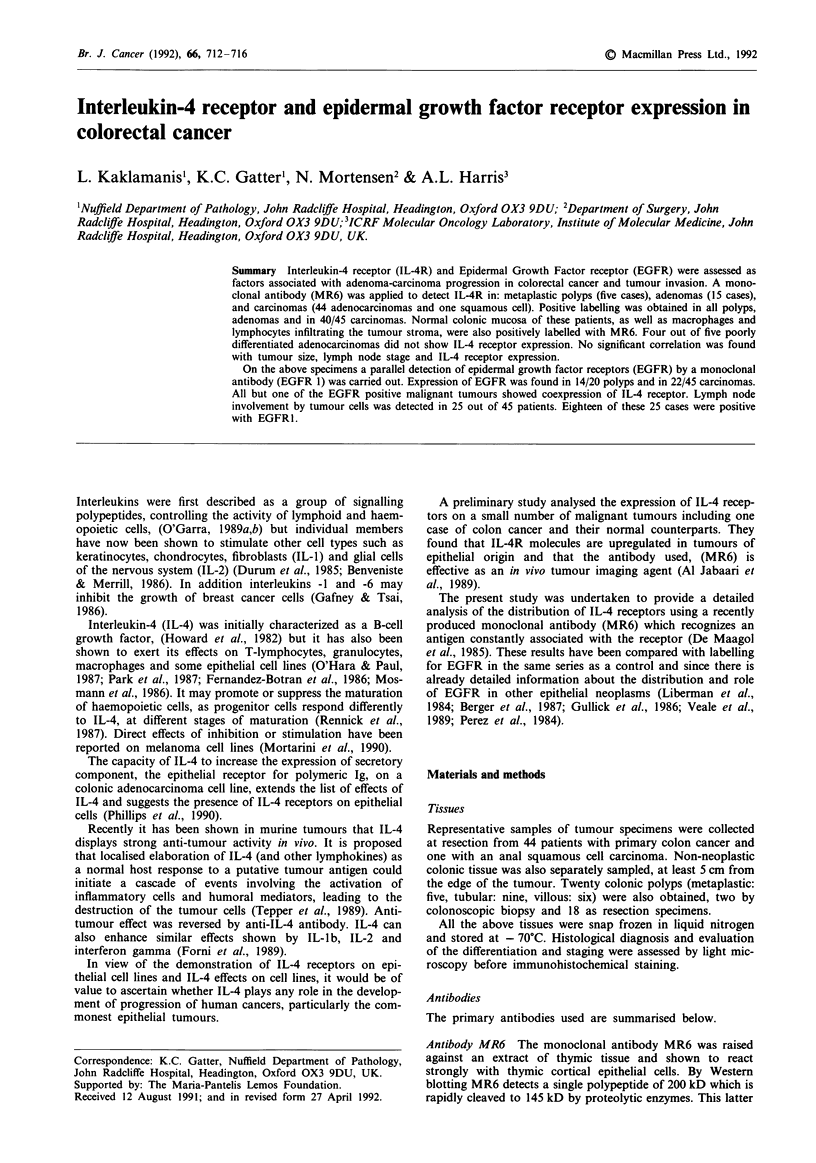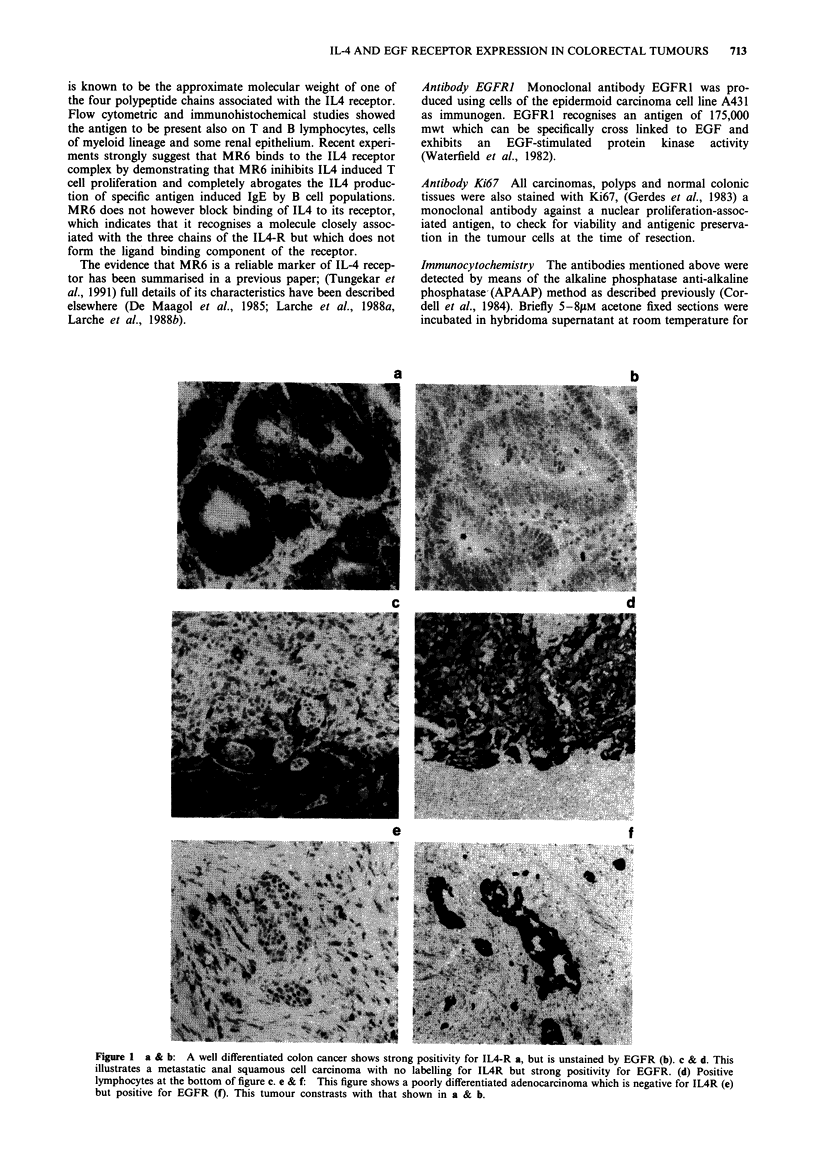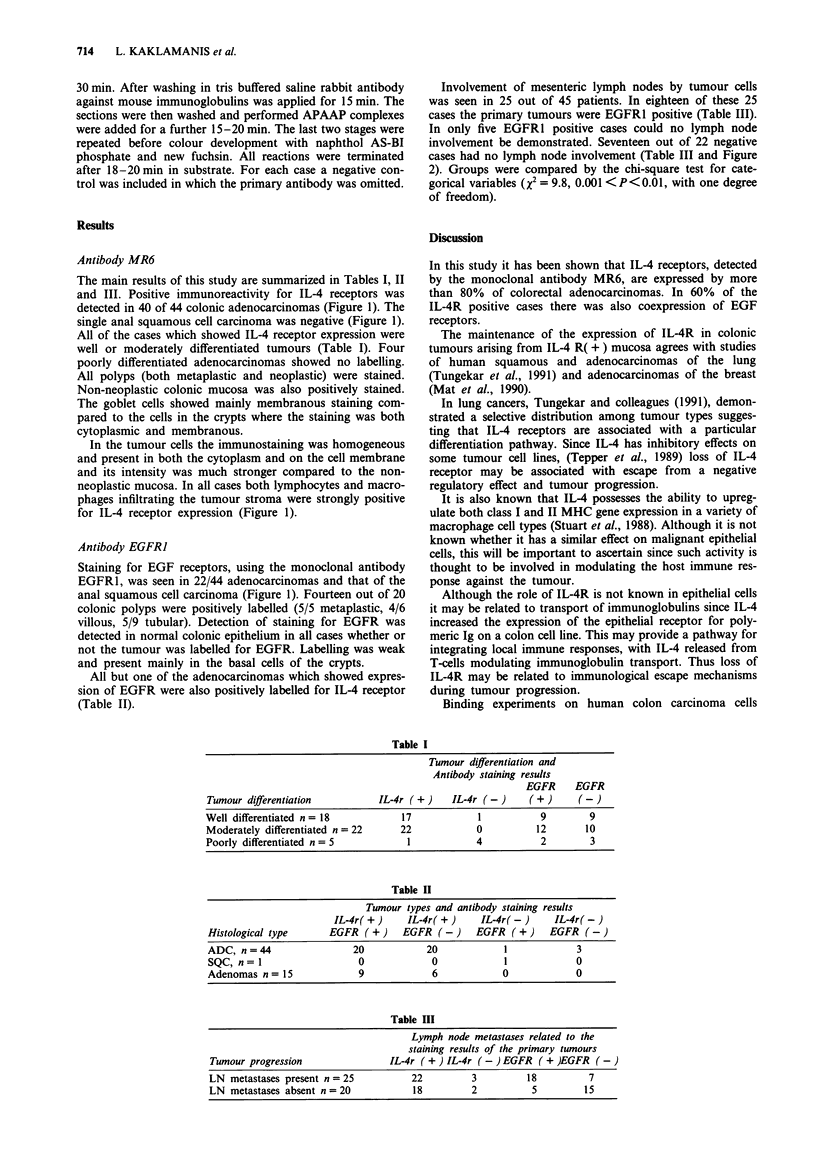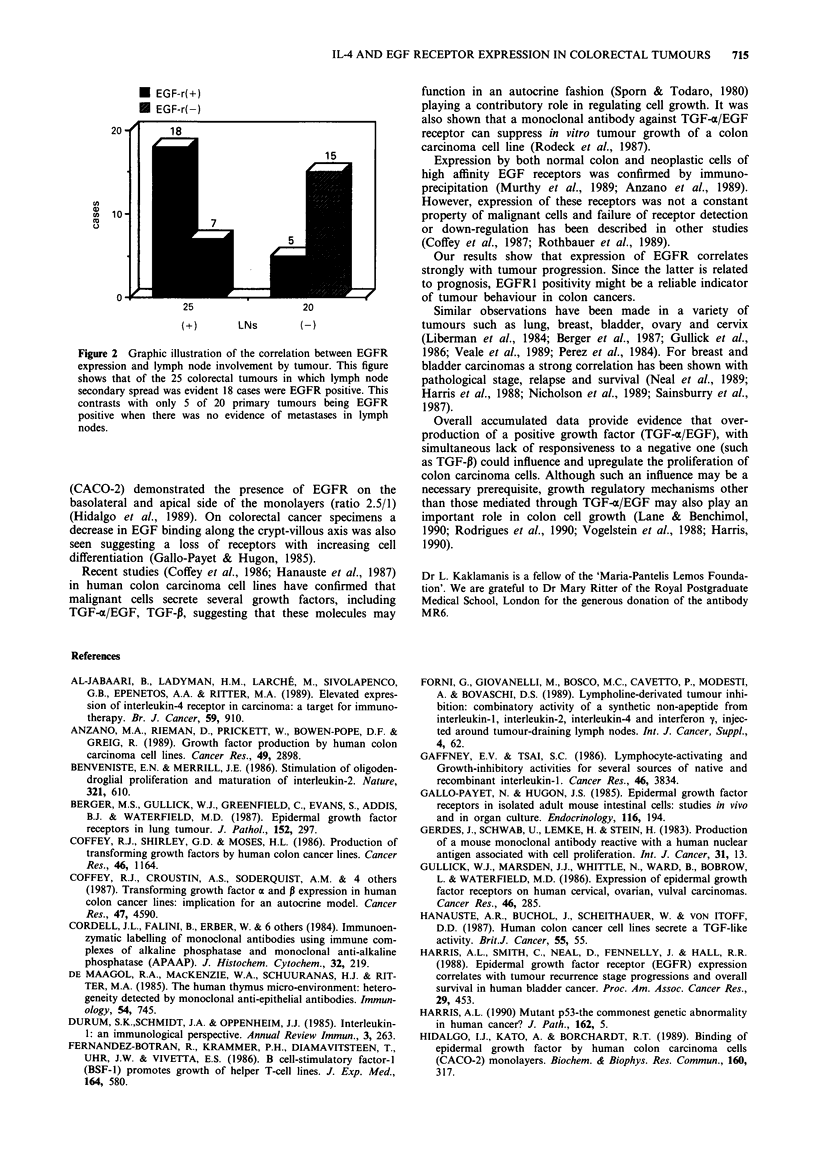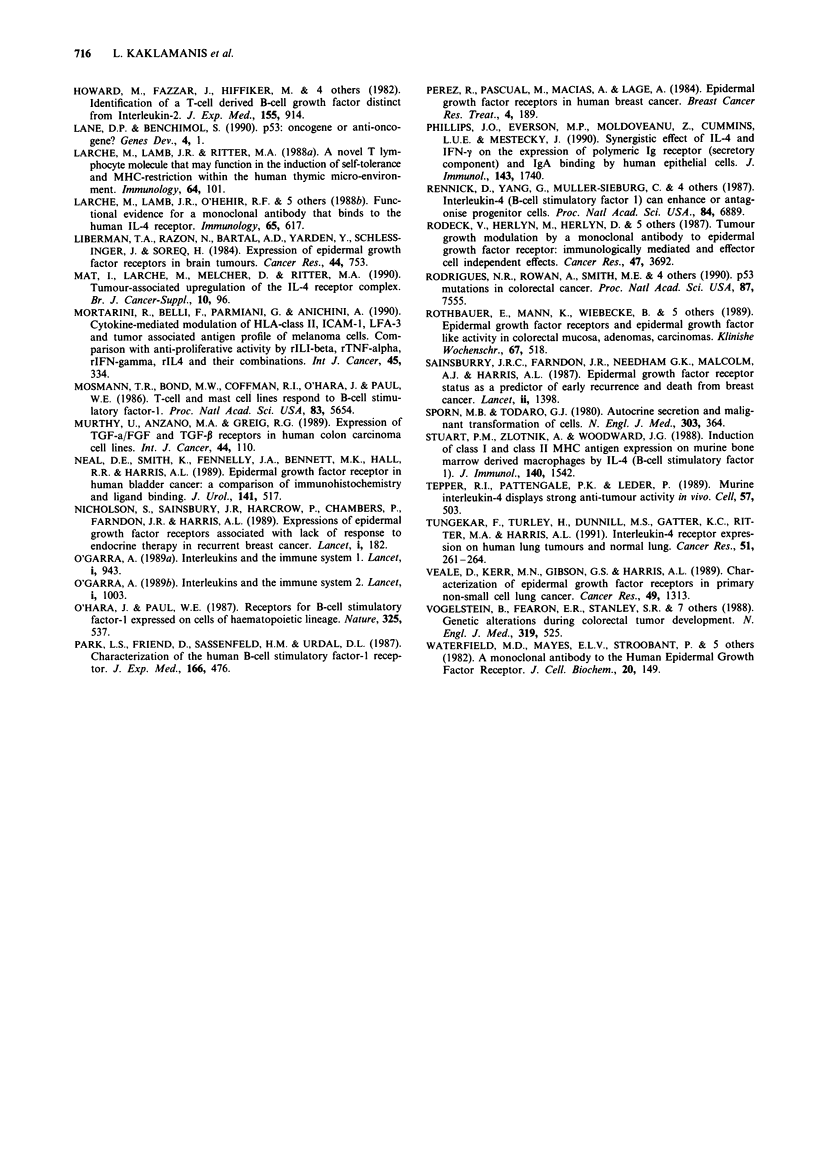# Interleukin-4 receptor and epidermal growth factor receptor expression in colorectal cancer.

**DOI:** 10.1038/bjc.1992.343

**Published:** 1992-10

**Authors:** L. Kaklamanis, K. C. Gatter, N. Mortensen, A. L. Harris

**Affiliations:** Nuffield Department of Pathology, John Radcliffe Hospital, Headington, Oxford, UK.

## Abstract

**Images:**


Br. J. Cancer (1992), 66, 712-716 © Macmillan Press Ltd., 1992

### Interleukin-4 receptor and epidermal growth factor receptor expression in colorectal cancer

L. Kaklamanis[1], K.C. Gatter[1], N. Mortensen[2] & A.L. Harris[3]

[1]*Nuffield Department of Pathology, John Radcliffe Hospital, Headington, Oxford OX3 9DU;* [2]*Department of Surgery, John Radcliffe Hospital, Headington, Oxford OX3 9DU;* [3]*ICRF Molecular Oncology Laboratory, Institute of Molecular Medicine, John Radcliffe Hospital, Headington, Oxford OX3 9DU, UK.*

**Summary** Interleukin-4 receptor (IL-4R) and Epidermal Growth Factor receptor (EGFR) were assessed as factors associated with adenoma-carcinoma progression in colorectal cancer and tumour invasion. A monoclonal antibody (MR6) was applied to detect IL-4R in: metaplastic polyps (five cases), adenomas (15 cases), and carcinomas (44 adenocarcinomas and one squamous cell). Positive labelling was obtained in all polyps, adenomas and in 40/45 carcinomas. Normal colonic mucosa of these patients, as well as macrophages and lymphocytes infiltrating the tumour stroma, were also positively labelled with MR6. Four out of five poorly differentiated adenocarcinomas did not show IL-4 receptor expression. No significant correlation was found with tumour size, lymph node stage and IL-4 receptor expression.

On the above specimens a parallel detection of epidermal growth factor receptors (EGFR) by a monoclonal antibody (EGFR 1) was carried out. Expression of EGFR was found in 14/20 polyps and in 22/45 carcinomas. All but one of the EGFR positive malignant tumours showed coexpression of IL-4 receptor. Lymph node involvement by tumour cells was detected in 25 out of 45 patients. Eighteen of these 25 cases were positive with EGFR1.

Interleukins were first described as a group of signalling polypeptides, controlling the activity of lymphoid and haemopoietic cells, (O'Garra, 1989*a,b*) but individual members have now been shown to stimulate other cell types such as keratinocytes, chondrocytes, fibroblasts (IL-1) and glial cells of the nervous system (IL-2) (Durum *et al.*, 1985; Benveniste & Merrill, 1986). In addition interleukins -1 and -6 may inhibit the growth of breast cancer cells (Gafney & Tsai, 1986).

Interleukin-4 (IL-4) was initially characterized as a B-cell growth factor, (Howard *et al.*, 1982) but it has also been shown to exert its effects on T-lymphocytes, granulocytes, macrophages and some epithelial cell lines (O'Hara & Paul, 1987; Park *et al.*, 1987; Fernandez-Botran *et al.*, 1986; Mosmann *et al.*, 1986). It may promote or suppress the maturation of haemopoietic cells, as progenitor cells respond differently to IL-4, at different stages of maturation (Rennick *et al.*, 1987). Direct effects of inhibition or stimulation have been reported on melanoma cell lines (Mortarini *et al.*, 1990).

The capacity of IL-4 to increase the expression of secretory component, the epithelial receptor for polymeric Ig, on a colonic adenocarcinoma cell line, extends the list of effects of IL-4 and suggests the presence of IL-4 receptors on epithelial cells (Phillips *et al.*, 1990).

Recently it has been shown in murine tumours that IL-4 displays strong anti-tumour activity *in vivo*. It is proposed that localised elaboration of IL-4 (and other lymphokines) as a normal host response to a putative tumour antigen could initiate a cascade of events involving the activation of inflammatory cells and humoral mediators, leading to the destruction of the tumour cells (Tepper *et al.*, 1989). Anti-tumour effect was reversed by anti-IL-4 antibody. IL-4 can also enhance similar effects shown by IL-1b, IL-2 and interferon gamma (Forni *et al.*, 1989).

In view of the demonstration of IL-4 receptors on epithelial cell lines and IL-4 effects on cell lines, it would be of value to ascertain whether IL-4 plays any role in the development of progression of human cancers, particularly the commonest epithelial tumours.

Correspondence: K.C. Gatter, Nuffield Department of Pathology, John Radcliffe Hospital, Headington, Oxford OX3 9DU, UK.
Supported by: The Maria-Pantelis Lemos Foundation.
Received 12 August 1991; and in revised form 27 April 1992.

A preliminary study analysed the expression of IL-4 receptors on a small number of malignant tumours including one case of colon cancer and their normal counterparts. They found that IL-4R molecules are upregulated in tumours of epithelial origin and that the antibody used, (MR6) is effective as an *in vivo* tumour imaging agent (Al Jabaari *et al.*, 1989).

The present study was undertaken to provide a detailed analysis of the distribution of IL-4 receptors using a recently produced monoclonal antibody (MR6) which recognizes an antigen constantly associated with the receptor (De Maagol *et al.*, 1985). These results have been compared with labelling for EGFR in the same series as a control and since there is already detailed information about the distribution and role of EGFR in other epithelial neoplasms (Liberman *et al.*, 1984; Berger *et al.*, 1987; Gullick *et al.*, 1986; Veale *et al.*, 1989; Perez *et al.*, 1984).

### Materials and methods

### *Tissues*

Representative samples of tumour specimens were collected at resection from 44 patients with primary colon cancer and one with an anal squamous cell carcinoma. Non-neoplastic colonic tissue was also separately sampled, at least 5 cm from the edge of the tumour. Twenty colonic polyps (metaplastic: five, tubular: nine, villous: six) were also obtained, two by colonoscopic biopsy and 18 as resection specimens.

All the above tissues were snap frozen in liquid nitrogen and stored at − 70°C. Histological diagnosis and evaluation of the differentiation and staging were assessed by light microscopy before immunohistochemical staining.

### *Antibodies*

The primary antibodies used are summarised below.

*Antibody MR6* The monoclonal antibody MR6 was raised against an extract of thymic tissue and shown to react strongly with thymic cortical epithelial cells. By Western blotting MR6 detects a single polypeptide of 200 kD which is rapidly cleaved to 145 kD by proteolytic enzymes. This latter is known to be the approximate molecular weight of one of the four polypeptide chains associated with the IL4 receptor. Flow cytometric and immunohistochemical studies showed the antigen to be present also on T and B lymphocytes, cells of myeloid lineage and some renal epithelium. Recent experiments strongly suggest that MR6 binds to the IL4 receptor complex by demonstrating that MR6 inihibits IL4 induced T cell proliferation and completely abrogates the IL4 production of specific antigen induced IgE by B cell populations. MR6 does not however block binding of IL4 to its receptor, which indicates that it recognises a molecule closely associated with the three chains of the IL4-R but which does not form the ligand binding component of the receptor.

The evidence that MR6 is a reliable marker of IL-4 receptor has been summarised in a previous paper; (Tungekar *et al.*, 1991) full details of its characteristics have been described elsewhere (De Maagol *et al.*, 1985; Larche *et al.*, 1988*a*, Larche *et al.*, 1988*b*).

*Antibody EGFR1* Monoclonal antibody EGFR1 was produced using cells of the epidermoid carcinoma cell line A431 as immunogen. EGFR1 recognises an antigen of 175,000 mwt which can be specifically cross linked to EGF and exhibits an EGF-stimulated protein kinase activity (Waterfield *et al.*, 1982).

*Antibody Ki67* All carcinomas, polyps and normal colonic tissues were also stained with Ki67, (Gerdes *et al.*, 1983) a monoclonal antibody against a nuclear proliferation-associated antigen, to check for viability and antigenic preservation in the tumour cells at the time of resection.

*Immunocytochemistry* The antibodies mentioned above were detected by means of the alkaline phosphatase anti-alkaline phosphatase (APAAP) method as described previously (Cordell *et al.*, 1984). Briefly 5–8μM acetone fixed sections were incubated in hybridoma supernatant at room temperature for

**Figure 1** **a & b**: A well differentiated colon cancer shows strong positivity for IL4-R **a**, but is unstained by EGFR (**b**). **c & d**. This illustrates a metastatic anal squamous cell carcinoma with no labelling for IL4R but strong positivity for EGFR. (**d**) Positive lymphocytes at the bottom of figure **c**. **e & f**: This figure shows a poorly differentiated adenocarcinoma which is negative for IL4R (**e**) but positive for EGFR (**f**). This tumour constrasts with that shown in **a & b**.

30 min. After washing in tris buffered saline rabbit antibody against mouse immunoglobulins was applied for 15 min. The sections were then washed and performed APAAP complexes were added for a further 15–20 min. The last two stages were repeated before colour development with naphthol AS-BI phosphate and new fuchsin. All reactions were terminated after 18–20 min in substrate. For each case a negative control was included in which the primary antibody was omitted.

### Results

### Antibody MR6

The main results of this study are summarized in Tables I, II and III. Positive immunoreactivity for IL-4 receptors was detected in 40 of 44 colonic adenocarcinomas (Figure 1). The single anal squamous cell carcinoma was negative (Figure 1). All of the cases which showed IL-4 receptor expression were well or moderately differentiated tumours (Table I). Four poorly differentiated adenocarcinomas showed no labelling. All polyps (both metaplastic and neoplastic) were stained. Non-neoplastic colonic mucosa was also positively stained. The goblet cells showed mainly membranous staining compared to the cells in the crypts where the staining was both cytoplasmic and membranous.

In the tumour cells the immunostaining was homogeneous and present in both the cytoplasm and on the cell membrane and its intensity was much stronger compared to the non-neoplastic mucosa. In all cases both lymphocytes and macrophages infiltrating the tumour stroma were strongly positive for IL-4 receptor expression (Figure 1).

### Antibody EGFR1

Staining for EGF receptors, using the monoclonal antibody EGFR1, was seen in 22/44 adenocarcinomas and that of the anal squamous cell carcinoma (Figure 1). Fourteen out of 20 colonic polyps were positively labelled (5/5 metaplastic, 4/6 villous, 5/9 tubular). Detection of staining for EGFR was detected in normal colonic epithelium in all cases whether or not the tumour was labelled for EGFR. Labelling was weak and present mainly in the basal cells of the crypts.

All but one of the adenocarcinomas which showed expression of EGFR were also positively labelled for IL-4 receptor (Table II).

Involvement of mesenteric lymph nodes by tumour cells was seen in 25 out of 45 patients. In eighteen of these 25 cases the primary tumours were EGFR1 positive (Table III). In only five EGFR1 positive cases could no lymph node involvement be demonstrated. Seventeen out of 22 negative cases had no lymph node involvement (Table III and Figure 2). Groups were compared by the chi-square test for categorical variables ($\chi^2 = 9.8$, $0.001 < P < 0.01$, with one degree of freedom).

### Discussion

In this study it has been shown that IL-4 receptors, detected by the monoclonal antibody MR6, are expressed by more than 80% of colorectal adenocarcinomas. In 60% of the IL-4R positive cases there was also coexpression of EGF receptors.

The maintenance of the expression of IL-4R in colonic tumours arising from IL-4 R( + ) mucosa agrees with studies of human squamous and adenocarcinomas of the lung (Tungekar *et al.*, 1991) and adenocarcinomas of the breast (Mat *et al.*, 1990).

In lung cancers, Tungekar and colleagues (1991), demonstrated a selective distribution among tumour types suggesting that IL-4 receptors are associated with a particular differentiation pathway. Since IL-4 has inhibitory effects on some tumour cell lines, (Tepper *et al.*, 1989) loss of IL-4 receptor may be associated with escape from a negative regulatory effect and tumour progression.

It is also known that IL-4 possesses the ability to upregulate both class I and II MHC gene expression in a variety of macrophage cell types (Stuart *et al.*, 1988). Although it is not known whether it has a similar effect on malignant epithelial cells, this will be important to ascertain since such activity is thought to be involved in modulating the host immune response against the tumour.

Although the role of IL-4R is not known in epithelial cells it may be related to transport of immunoglobulins since IL-4 increased the expression of the epithelial receptor for polymeric Ig on a colon cell line. This may provide a pathway for integrating local immune responses, with IL-4 released from T-cells modulating immunoglobulin transport. Thus loss of IL-4R may be related to immunological escape mechanisms during tumour progression.

Binding experiments on human colon carcinoma cells

**Table I**

| | *Tumour differentiation and Antibody staining results* | | | |
|---|---|---|---|---|
| *Tumour differentiation* | *IL-4r ( + )* | *IL-4r ( − )* | *EGFR ( + )* | *EGFR ( − )* |
| Well differentiated $n = 18$ | 17 | 1 | 9 | 9 |
| Moderately differentiated $n = 22$ | 22 | 0 | 12 | 10 |
| Poorly differentiated $n = 5$ | 1 | 4 | 2 | 3 |

**Table II**

| | *Tumour types and antibody staining results* | | | |
|---|---|---|---|---|
| *Histological type* | *IL-4r( + ) EGFR ( + )* | *IL-4r( + ) EGFR ( − )* | *IL-4r( − ) EGFR ( + )* | *IL-4r( − ) EGFR ( − )* |
| ADC, $n = 44$ | 20 | 20 | 1 | 3 |
| SQC, $n = 1$ | 0 | 0 | 1 | 0 |
| Adenomas $n = 15$ | 9 | 6 | 0 | 0 |

**Table III**

| | *Lymph node metastases related to the staining results of the primary tumours* | | | |
|---|---|---|---|---|
| *Tumour progression* | *IL-4r ( + )* | *IL-4r ( − )* | *EGFR ( + )* | *EGFR ( − )* |
| LN metastases present $n = 25$ | 22 | 3 | 18 | 7 |
| LN metastases absent $n = 20$ | 18 | 2 | 5 | 15 |

**Figure 2** Graphic illustration of the correlation between EGFR expression and lymph node involvement by tumour. This figure shows that of the 25 colorectal tumours in which lymph node secondary spread was evident 18 cases were EGFR positive. This contrasts with only 5 of 20 primary tumours being EGFR positive when there was no evidence of metastases in lymph nodes.

(CACO-2) demonstrated the presence of EGFR on the basolateral and apical side of the monolayers (ratio 2.5/1) (Hidalgo *et al.*, 1989). On colorectal cancer specimens a decrease in EGF binding along the crypt-villous axis was also seen suggesting a loss of receptors with increasing cell differentiation (Gallo-Payet & Hugon, 1985).

Recent studies (Coffey *et al.*, 1986; Hanauste *et al.*, 1987) in human colon carcinoma cell lines have confirmed that malignant cells secrete several growth factors, including TGF-$\alpha$/EGF, TGF-$\beta$, suggesting that these molecules may function in an autocrine fashion (Sporn & Todaro, 1980) playing a contributory role in regulating cell growth. It was also shown that a monoclonal antibody against TGF-$\alpha$/EGF receptor can suppress *in vitro* tumour growth of a colon carcinoma cell line (Rodeck *et al.*, 1987).

Expression by both normal colon and neoplastic cells of high affinity EGF receptors was confirmed by immunoprecipitation (Murthy *et al.*, 1989; Anzano *et al.*, 1989). However, expression of these receptors was not a constant property of malignant cells and failure of receptor detection or down-regulation has been described in other studies (Coffey *et al.*, 1987; Rothbauer *et al.*, 1989).

Our results show that expression of EGFR correlates strongly with tumour progression. Since the latter is related to prognosis, EGFR1 positivity might be a reliable indicator of tumour behaviour in colon cancers.

Similar observations have been made in a variety of tumours such as lung, breast, bladder, ovary and cervix (Liberman *et al.*, 1984; Berger *et al.*, 1987; Gullick *et al.*, 1986; Veale *et al.*, 1989; Perez *et al.*, 1984). For breast and bladder carcinomas a strong correlation has been shown with pathological stage, relapse and survival (Neal *et al.*, 1989; Harris *et al.*, 1988; Nicholson *et al.*, 1989; Sainsbury *et al.*, 1987).

Overall accumulated data provide evidence that overproduction of a positive growth factor (TGF-$\alpha$/EGF), with simultaneous lack of responsiveness to a negative one (such as TGF-$\beta$) could influence and upregulate the proliferation of colon carcinoma cells. Although such an influence may be a necessary prerequisite, growth regulatory mechanisms other than those mediated through TGF-$\alpha$/EGF may also play an important role in colon cell growth (Lane & Benchimol, 1990; Rodrigues *et al.*, 1990; Vogelstein *et al.*, 1988; Harris, 1990).

Dr L. Kaklamanis is a fellow of the 'Maria-Pantelis Lemos Foundation'. We are grateful to Dr Mary Ritter of the Royal Postgraduate Medical School, London for the generous donation of the antibody MR6.

### References

AL-JABAARI, B., LADYMAN, H.M., LARCHÉ, M., SIVOLAPENCO, G.B., EPENETOS, A.A. & RITTER, M.A. (1989). Elevated expression of interleukin-4 receptor in carcinoma: a target for immunotherapy. *Br. J. Cancer*, **59**, 910.

ANZANO, M.A., RIEMAN, D., PRICKETT, W., BOWEN-POPE, D.F. & GREIG, R. (1989). Growth factor production by human colon carcinoma cell lines. *Cancer Res.*, **49**, 2898.

BENVENISTE, E.N. & MERRILL, J.E. (1986). Stimulation of oligodendroglial proliferation and maturation of interleukin-2. *Nature*, **321**, 610.

BERGER, M.S., GULLICK, W.J., GREENFIELD, C., EVANS, S., ADDIS, B.J. & WATERFIELD, M.D. (1987). Epidermal growth factor receptors in lung tumour. *J. Pathol.*, **152**, 297.

COFFEY, R.J., SHIRLEY, G.D. & MOSES, H.L. (1986). Production of transforming growth factors by human colon cancer lines. *Cancer Res.*, **46**, 1164.

COFFEY, R.J., CROUSTIN, A.S., SODERQUIST, A.M. & 4 others (1987). Transforming growth factor $\alpha$ and $\beta$ expression in human colon cancer lines: implication for an autocrine model. *Cancer Res.*, **47**, 4590.

CORDELL, J.L., FALINI, B., ERBER, W. & 6 others (1984). Immunoenzymatic labelling of monoclonal antibodies using immune complexes of alkaline phosphatase and monoclonal anti-alkaline phosphatase (APAAP). *J. Histochem. Cytochem.*, **32**, 219.

DE MAAGOL, R.A., MACKENZIE, W.A., SCHUURANAS, H.J. & RITTER, M.A. (1985). The human thymus micro-environment: heterogeneity detected by monoclonal anti-epithelial antibodies. *Immunology*, **54**, 745.

DURUM, S.K.,SCHMIDT, J.A. & OPPENHEIM, J.J. (1985). Interleukin-1: an immunological perspective. *Annual Review Immun.*, **3**, 263.

FERNANDEZ-BOTRAN, R., KRAMMER, P.H., DIAMAVITSTEEN, T., UHR, J.W. & VIVETTA, E.S. (1986). B cell-stimulatory factor-1 (BSF-1) promotes growth of helper T-cell lines. *J. Exp. Med.*, **164**, 580.

FORNI, G., GIOVANELLI, M., BOSCO, M.C., CAVETTO, P., MODESTI, A. & BOVASCHI, D.S. (1989). Lympholine-derivated tumour inhibition: combinatory activity of a synthetic non-apeptide from interleukin-1, interleukin-2, interleukin-4 and interferon $\gamma$, injected around tumour-draining lymph nodes. *Int. J. Cancer, Suppl.*, **4**, 62.

GAFFNEY, E.V. & TSAI, S.C. (1986). Lymphocyte-activating and Growth-inhibitory activities for several sources of native and recombinant interleukin-1. *Cancer Res.*, **46**, 3834.

GALLO-PAYET, N. & HUGON, J.S. (1985). Epidermal growth factor receptors in isolated adult mouse intestinal cells: studies *in vivo* and in organ culture. *Endocrinology*, **116**, 194.

GERDES, J., SCHWAB, U., LEMKE, H. & STEIN, H. (1983). Production of a mouse monoclonal antibody reactive with a human nuclear antigen associated with cell proliferation. *Int. J. Cancer*, **31**, 13.

GULLICK, W.J., MARSDEN, J.J., WHITTLE, N., WARD, B., BOBROW, L. & WATERFIELD, M.D. (1986). Expression of epidermal growth factor receptors on human cervical, ovarian, vulval carcinomas. *Cancer Res.*, **46**, 285.

HANAUSTE, A.R., BUCHOL, J., SCHEITHAUER, W. & VON ITOFF, D.D. (1987). Human colon cancer cell lines secrete a TGF-like activity. *Brit.J. Cancer*, **55**, 55.

HARRIS, A.L., SMITH, C., NEAL, D., FENNELLY, J. & HALL, R.R. (1988). Epidermal growth factor receptor (EGFR) expression correlates with tumour recurrence stage progressions and overall survival in human bladder cancer. *Proc. Am. Assoc. Cancer Res.*, **29**, 453.

HARRIS, A.L. (1990) Mutant p53-the commonest genetic abnormality in human cancer? *J. Path.*, **162**, 5.

HIDALGO, I.J., KATO, A. & BORCHARDT, R.T. (1989). Binding of epidermal growth factor by human colon carcinoma cells (CACO-2) monolayers. *Biochem. & Biophys. Res. Commun.*, **160**, 317.

HOWARD, M., FAZZAR, J., HIFFIKER, M. & 4 others (1982). Identification of a T-cell derived B-cell growth factor distinct from Interleukin-2. *J. Exp. Med.*, **155**, 914.

LANE, D.P. & BENCHIMOL, S. (1990). p53: oncogene or anti-oncogene? *Genes Dev.*, **4**, 1.

LARCHE, M., LAMB, J.R. & RITTER, M.A. (1988*a*). A novel T lymphocyte molecule that may function in the induction of self-tolerance and MHC-restriction within the human thymic micro-environment. *Immunology*, **64**, 101.

LARCHE, M., LAMB, J.R., O'HEHIR, R.F. & 5 others (1988*b*). Functional evidence for a monoclonal antibody that binds to the human IL-4 receptor. *Immunology*, **65**, 617.

LIBERMAN, T.A., RAZON, N., BARTAL, A.D., YARDEN, Y., SCHLESSINGER, J. & SOREQ, H. (1984). Expression of epidermal growth factor receptors in brain tumours. *Cancer Res.*, **44**, 753.

MAT, I., LARCHE, M., MELCHER, D. & RITTER, M.A. (1990). Tumour-associated upregulation of the IL-4 receptor complex. *Br. J. Cancer-Suppl.*, **10**, 96.

MORTARINI, R., BELLI, F., PARMIANI, G. & ANICHINI, A. (1990). Cytokine-mediated modulation of HLA-class II, ICAM-1, LFA-3 and tumor associated antigen profile of melanoma cells. Comparison with anti-proliferative activity by rILI-beta, rTNF-alpha, rIFN-gamma, rIL4 and their combinations. *Int J. Cancer*, **45**, 334.

MOSMANN, T.R., BOND, M.W., COFFMAN, R.I., O'HARA, J. & PAUL, W.E. (1986). T-cell and mast cell lines respond to B-cell stimulatory factor-1. *Proc. Natl Acad. Sci. USA*, **83**, 5654.

MURTHY, U., ANZANO, M.A. & GREIG, R.G. (1989). Expression of TGF-a/FGF and TGF-$\beta$ receptors in human colon carcinoma cell lines. *Int. J. Cancer*, **44**, 110.

NEAL, D.E., SMITH, K., FENNELLY, J.A., BENNETT, M.K., HALL, R.R. & HARRIS, A.L. (1989). Epidermal growth factor receptor in human bladder cancer: a comparison of immunohistochemistry and ligand binding. *J. Urol.*, **141**, 517.

NICHOLSON, S., SAINSBURY, J.R, HARCROW, P., CHAMBERS, P., FARNDON, J.R. & HARRIS, A.L. (1989). Expressions of epidermal growth factor receptors associated with lack of response to endocrine therapy in recurrent breast cancer. *Lancet*, **i**, 182.

O'GARRA, A. (1989*a*). Interleukins and the immune system 1. *Lancet*, **i**, 943.

O'GARRA, A. (1989*b*). Interleukins and the immune system 2. *Lancet*, **i**, 1003.

O'HARA, J. & PAUL, W.E. (1987). Receptors for B-cell stimulatory factor-1 expressed on cells of haematopoietic lineage. *Nature*, **325**, 537.

PARK, L.S., FRIEND, D., SASSENFELD, H.M. & URDAL, D.L. (1987). Characterization of the human B-cell stimulatory factor-1 receptor. *J. Exp. Med.*, **166**, 476.

PEREZ, R., PASCUAL, M., MACIAS, A. & LAGE, A. (1984). Epidermal growth factor receptors in human breast cancer. *Breast Cancer Res. Treat.*, **4**, 189.

PHILLIPS, J.O., EVERSON, M.P., MOLDOVEANU, Z., CUMMINS, L.U.E. & MESTECKY, J. (1990). Synergistic effect of IL-4 and IFN-$\gamma$ on the expression of polymeric Ig receptor (secretory component) and IgA binding by human epithelial cells. *J. Immunol.*, **143**, 1740.

RENNICK, D., YANG, G., MULLER-SIEBURG, C. & 4 others (1987). Interleukin-4 (B-cell stimulatory factor 1) can enhance or antagonise progenitor cells. *Proc. Natl Acad. Sci. USA.*, **84**, 6889.

RODECK, V., HERLYN, M., HERLYN, D. & 5 others (1987). Tumour growth modulation by a monoclonal antibody to epidermal growth factor receptor: immunologically mediated and effector cell independent effects. *Cancer Res.*, **47**, 3692.

RODRIGUES, N.R., ROWAN, A., SMITH, M.E. & 4 others (1990). p53 mutations in colorectal cancer. *Proc. Natl Acad. Sci. USA*, **87**, 7555.

ROTHBAUER, E., MANN, K., WIEBECKE, B. & 5 others (1989). Epidermal growth factor receptors and epidermal growth factor like activity in colorectal mucosa, adenomas, carcinomas. *Klinishe Wochenschr.*, **67**, 518.

SAINSBURRY, J.R.C., FARNDON, J.R., NEEDHAM G.K., MALCOLM, A.J. & HARRIS, A.L. (1987). Epidermal growth factor receptor status as a predictor of early recurrence and death from breast cancer. *Lancet*, **ii**, 1398.

SPORN, M.B. & TODARO, G.J. (1980). Autocrine secretion and malignant transformation of cells. *N. Engl. J. Med.*, **303**, 364.

STUART, P.M., ZLOTNIK, A. & WOODWARD, J.G. (1988). Induction of class I and class II MHC antigen expression on murine bone marrow derived macrophages by IL-4 (B-cell stimulatory factor 1). *J. Immunol.*, **140**, 1542.

TEPPER, R.I., PATTENGALE, P.K. & LEDER, P. (1989). Murine interleukin-4 displays strong anti-tumour activity *in vivo*. *Cell*, **57**, 503.

TUNGEKAR, F., TURLEY, H., DUNNILL, M.S., GATTER, K.C., RITTER, M.A. & HARRIS, A.L. (1991). Interleukin-4 receptor expression on human lung tumours and normal lung. *Cancer Res.*, **51**, 261–264.

VEALE, D., KERR, M.N., GIBSON, G.S. & HARRIS, A.L. (1989). Characterization of epidermal growth factor receptors in primary non-small cell lung cancer. *Cancer Res.*, **49**, 1313.

VOGELSTEIN, B., FEARON, E.R., STANLEY, S.R. & 7 others (1988). Genetic alterations during colorectal tumor development. *N. Engl. J. Med.*, **319**, 525.

WATERFIELD, M.D., MAYES, E.L.V., STROOBANT, P. & 5 others (1982). A monoclonal antibody to the Human Epidermal Growth Factor Receptor. *J. Cell. Biochem.*, **20**, 149.